# Rat epidermal stem cells promote the angiogenesis of full-thickness wounds

**DOI:** 10.1186/s13287-020-01844-y

**Published:** 2020-08-08

**Authors:** Shaobin Huang, Zhicheng Hu, Peng Wang, Yi Zhang, Xiaoling Cao, Yunxian Dong, Pu Cheng, Hailin Xu, Wenkai Zhu, Bing Tang, Jiayuan Zhu

**Affiliations:** 1 Department of Burn, First Affiliated Hospital of Sun Yat-sen University, Guangzhou, 510080 Guangdong Province People’s Republic of China; 2 Department of Plastic Surgery, Sixth Affiliated Hospital of Sun Yat-sen University, Guangzhou, 510080 Guangdong Province People’s Republic of China; 3 Department of Burn and Plastic Surgery, Affiliated Hospital of Nantong University, Nantong, People’s Republic of China; 4 Department of Chemistry, Portland State University, Portland, USA

**Keywords:** ESCs, Cell differentiation, Angiogenesis, Full-thickness wounds

## Abstract

**Background:**

Full-thickness wounds severely affect patients’ life quality and become challenging problems for clinicians. Stem cells have great prospects in the treatment of wounds. Our previous study confirmed that autologous basal cell suspension could promote wound healing, and epidermal stem cells (ESCs) were detected in the basal cell suspension. Herein, this study aimed to explore the effect of ESCs on full-thickness wounds.

**Methods:**

Rat ESCs were isolated and expanded and then were transfected with lentivirus to stably express enhanced green fluorescent protein. The experimental rats were randomly divided into 2 groups: in the ESC group, the rat ESCs were sprayed on the full-thickness wounds of rats; in the control group, phosphate-buffered saline was sprayed the on the wounds. Next, wound healing and neovascularization were evaluated. Colonization, division, and differentiation of ESCs on the wound were analyzed by immunofluorescence.

**Results:**

The rat ESCs colonized, divided, and proliferated in the wound. Additionally, rat ESCs around blood vessels differentiated into vascular endothelial cells and formed a lumen-like structure. Compared with the control group, the ESC group showed enhanced angiogenesis and accelerated wound healing.

**Conclusions:**

Our study confirmed that rat ESCs are safe and effective for treating full-thickness wounds. Additionally, under certain conditions, ESCs can differentiate into vascular endothelial cells to promote angiogenesis and wound healing.

## Background

The skin is the largest organ of the human body and has many important functions, such as metabolism, absorption, protection, body temperature regulation, secretion, and sensation. At the same time, it is easily damaged when stimulated by external chemical and physical factors. Full-thickness skin wounds caused by varicose veins, arterial occlusion, burns, car accident injuries, war injuries, avulsion injuries, and other traumas are the most common conditions in clinical emergency departments and have always posed difficult problems for clinicians [1, 2]. In patients with full-thickness skin defects, the wounds are healed mainly by the migration of stem cells adjacent to the skin epithelial cells and regeneration of the remaining skin appendages. If the area of the patient’s skin defect is too large, it may take much longer time to heal, causing follow-up problems, such as scars and wound contraction, which affected normal skin function, esthetics, and psychology. Thus, accelerating the speed of repair after skin injury has become the focus of attention of clinicians. Presently, wound repair treatment mainly relies on surgical debridement, skin flap covering, skin grafting, wound dressing, application of epidermal factors, and hyperbaric oxygen therapy but lacks simple and effective methods [3–6]. Therefore, how to increase the effectiveness of wound healing and regain skin function are the problems faced by burn and plastic surgeons.

Effective wound repair requires the formation of many new blood vessels in the granulation tissue to maintain the nutrition of the wound bed and promote the deposition of extracellular matrix. Therefore, neovascularization in the wound tissue plays a very important role in forming granulation tissue, improving the microcirculation of the wound, reducing the incidence of infection, and promoting the healing of chronic refractory wounds or deep burn wounds. Impaired neovascularization will directly lead to delayed wound healing or poor wound healing [7–10].

Stem cells are considered ideal seed cells to promote wound healing due to their strong self-renewal capacity and multidirectional differentiation potential [11, 12]. Our previous studies demonstrated that autologous basal cell suspension could promote skin re-epithelialization and wound healing. Moreover, in the treatment of chronic wounds, blade thick-skin transplantation combined with autologous basal cell suspension can improve the survival rate of skin and increase the quality of skin healing. The number of epidermal stem cells (ESCs) is very small (accounting for 1% to 10% of epidermal basal cells) [13–16]. We speculated that the epidermal stem cells might play an important role in wound healing. Thus, this study aimed to study the effects of epidermal stem cells on wound healing.

## Methods

### Isolation of rat ESCs

One six-week-old rat was sacrificed by cervical dislocation. The back skin of the rat was removed and placed in a 15-ml centrifuge tube with 1% phosphate-buffered saline (PBS; 10010023; Gibco) and then was placed on ice. The muscle layer was removed, and the skin was cut to a size of 1 × 1 cm^2^ and placed in a sterile 15-ml centrifuge tube. Next, 2 ml of × 10 Tryple (A1217702; Gibco) was added, and the sample was digested in a constant-temperature water bath at 37 °C for 15–30 min with shaking every 3 min. Several T25 culture flasks were evenly coated with 1 ml (0.5 mg/ml) of fibronectin (FN; ~ 5 μg/cm^2^; Shanghai Fibronectin Biotechnology, Shanghai, China) solution before planting the basal cell suspension and then were incubated in a 37 °C incubator for 20 min. After the skin was completely digested, it was rinsed with 1% PBS to stop the digestion. Next, the basal cells were scraped off using a sterile scalpel, rinsed, and collected into keratinocyte serum-free medium (K-SFM; 17005042; Gibco), and then the cell suspension was filtered in a 50-ml centrifuge tube using a 200-mesh filter. The cell suspension was transferred to a 15-ml centrifuge tube and centrifuged at 1000 r/min for 10 min. The supernatant was discarded, and the pellet was resuspended in 4 ml of complete medium by pipetting. The basal cell suspension was then used to coat the culture flask, which was then incubated in a 37 °C incubator for 20 min. Approximately 10% of the cells adhered to the wall first; these cells were regarded as ESCs. The ESCs were cultured in K-SFM medium at 37 °C, and the medium was changed every 2 days.

### Immunofluorescence and confocal microscopy

Cell passaging was performed when the cell density of the second generation of ESCs reached ~ 90%. Briefly, 1 ml of 0.25% trypsin was added to the flask, which was gently shaken so that the trypsin covered the cell surface evenly and then placed in a cell culture incubated for 3 min. After the cells dissociated from the flask wall, 3 ml of complete medium was added to stop digestion. The samples were then centrifuged at 1000 r/min for 5 min, the supernatants were removed, and then the pellets were resuspended and cultured in confocal-grade glass-bottomed Petri dishes. After the cells adhered, they were washed 3 times with PBS for 5 min each, fixed with 4% paraformaldehyde (P0099-500 ml; Beyotime Biotechnology, Shanghai, China) for 20 min, washed again with PBS 3 times, and then incubated with 0.5% Triton X-100 (P0096-500 ml; Beyotime Biotechnology, Shanghai, China) at room temperature for 20 min. The samples were washed with PBS three times, and then 5% goat serum blocking solution (C0265; Beyotime Biotechnology, Shanghai, China) was added to the glass-bottomed Petri dishes, followed by incubation at room temperature for 40 min. The blocking solution was aspirated, and then the samples were incubated at 4 °C overnight with 100 μl of the following diluted primary antibodies: p63 (1:200; ab735; Abcam), α6-integrin (1:200; ab235905; Abcam), CD71 (1:200; ab22391; Abcam), CK15 (1:200; ab80522; Abcam), CK19 (1:200; ab84632; Abcam), CD31 (1:200; ab24590; Abcam), and CD34 (1:200; ab81289; Abcam). Next, the samples were washed three times with PBS for 5 min each. After aspirating the excess liquid from the Petri dishes, the samples were incubated for 1 h at room temperature in the dark with the following diluted fluorescent secondary antibodies: goat anti-rabbit IgG labeled with Alex Fluor 488 (1:200; ab150077; Abcam), goat anti-mouse IgG labeled with Alexa Fluor 594 (1:200; ab150116; Abcam), and goat anti-rat IgG H&L DyLight® 594 (1:200; ab96889; Abcam). DAPI (D9542; Sigma-Aldrich) was added dropwise to the samples, followed by incubation for 5 min in the dark, and then the samples were rinsed 4 times with PBS for 5 min each. The supernatant was aspirated, and then the samples were mounted with an anti-fluorescence quencher and observed under a fluorescence microscope, followed by image analysis.

### Flow cytometry

Third-generation rat ESCs were collected by centrifugation, and the supernatant was aspirated. The cells were then resuspended in 0.5 ml of PBS and then were fixed and permeabilized using the BD Cytofix/Cytoperm™ Fixation/Permeabilization Kit. Next, 2–3 ml of incubation buffer was added to the cells, followed by rinsing by centrifugation. The cells were resuspended in 100 μl of incubation buffer per test tube and were blocked by incubation for 10 min at room temperature. Next, the primary antibodies (p63, Abcam, ab124762, 1:200; α6-integrin, Abcam, ab77906, 1:200) were added to the tubes at the appropriate dilution and incubated at room temperature for 60 min. Next, the cells were rinsed with incubation buffer by centrifugation. They then were incubated at room temperature for 30 min with fluorescent-labeled secondary antibodies diluted in incubation buffer as recommended by the manufacturer. Next, the cells were rinsed in incubation buffer by centrifugation, resuspended in 0.5 ml of PBS, and then subjected to flow cytometry analysis.

### Lentivirus and transfection

To generate ESCs with stable enhanced green fluorescent protein (EGFP) expression, the cells were infected with a lentiviral vector encoding the full-length human EGFP gene or empty lentiviral vector as the control (OBiO Technology, Shanghai, China). Stable clones were selected after 2 weeks using 1 μg/ml of puromycin, and the EGFP expression level was determined by immunofluorescence.

### Animal experiments

To explore the function of ESCs in full-thickness wound beds in vivo, the rat dorsal wound model was adopted. Twenty rats were anesthetized by inhaling isoflurane (INH), and a 2-cm-diameter, full-thickness wound was made on the dorsal skin of each rat. The wounds were divided into 2 groups randomly: control group and ESC group. ESCs that stably expressed EGFP were evenly sprayed on the wound bed using a 2-ml syringe. For the ESC group, 1 ml of cell suspension at a cell density of 1 × 10^5^/ml was evenly sprayed on the wound bed; for the control group, 1 ml of PBS was sprayed. The rats and wounds were observed, photographed, and measured daily until the rats were sacrificed. The wound healing time was recorded, and the residual wound area rate was calculated as [(day n area)/(day 0 area)] × 100% (*n* = 0, 3, 7, 14, or 21). Six rats of each group were sacrificed on days 0, 3, 7, 14, and 21, respectively, and the wound tissues were harvested and separated into two halves across the center: one half was processed for histological and immunohistochemistry analyses, and the other was rapidly frozen in liquid nitrogen for western blot analysis.

### Immunohistochemistry analysis

The paraffin-embedded fixed tissue sections of each group were deparaffinized and rehydrated in xylene and graded ethanol. Antigen retrieval was performed using Proteinase K solution (20 μg/ml) at 37 °C for 15 min. After blocking with Bloxall, the sections were blocked with goat serum for 30 min and then were incubated with primary antibody anti-CD31 (1:100; ab24590; Abcam) overnight at 4 °C. After washing in PBST, the sections were then incubated with an HRP conjugated secondary antibody (1:2000; ab97051; Abcam) for 1 h at room temperature. The sections were further incubated with 3,3′-diaminobenzidine (DAB) and counterstained with hematoxylin and observed by microscopy.

### Western blot analysis

Western blotting was performed using antibodies directed against CD31 (1:1000; ab24590; Abcam) and GAPDH (Sigma-Aldrich; SAB1405848; 1:6000). GAPDH served as an internal control. The cells were lysed with radioimmunoprecipitation assay (RIPA) lysis buffer (Cell Signaling Technology) containing PMSF (1:100; v/v) (Cell Signaling Technology) for 30 min. The BCA Protein Assay Kit (Pierce, Thermo Scientific) was used to measure the total protein concentrations. Aliquots (40 μg) of total cellular protein were resolved by SDS-PAGE (10~12%), electrotransferred to PVDF membranes, and blocked with 5% skim milk (w/v) at room temperature for 1 h. The membranes were then incubated with primary antibodies on an orbital shaker at 4 °C overnight, and secondary antibodies (HRP-conjugated goat anti-mouse and HRP-conjugated goat anti-rabbit) were added and incubated for 1 h at room temperature. Protein-antibody complexes were then detected by chemiluminescence (Pierce ECL Western Blotting Substrate, Thermo, USA).

### Tissue immunofluorescence analysis

Formalin-fixed and paraffin-embedded tissue sections were deparaffinized in xylene and rehydrated through a graded ethanol series. Antigen retrieval was performed using citrate buffer in a pressure cooker at 95 °C for 30 min. The 4-μm sections of each group were blocked in 10% goat serum (16210064; Gibco) for 30 min at 37 °C and then were incubated with the primary anti-rat CD31 antibody (Abcam; ab24590; 1:200). After incubating at 4 °C overnight, the sections were washed with PBST and incubated for 1 h with a goat anti-mouse IgG secondary antibody labeled with Alexa Fluor 594 (1:200; ab150116; Abcam). DAPI was added dropwise and incubated with the sections for 5 min in the dark, and then the sections were rinsed 4 times with PBS for 5 min each. The remaining liquid in the Petri dish was aspirated, and the sections were mounted with an anti-fluorescence quencher. The sections were analyzed by fluorescence microscopy (OLYMPUS, Japan).

### Statistical analysis

The values were expressed as means ± standard deviation (SD) unless otherwise indicated. Comparisons of the expression difference between the control and experimental groups were conducted by Student’s *t* test. All statistical analyses were performed using SPSS 20.0 software (SPSS, Chicago, IL, USA), and *P* < 0.05 indicated that the difference was statistically significant.

## Results

### Morphology and identification of rat ESCs

**Fig. 1:**
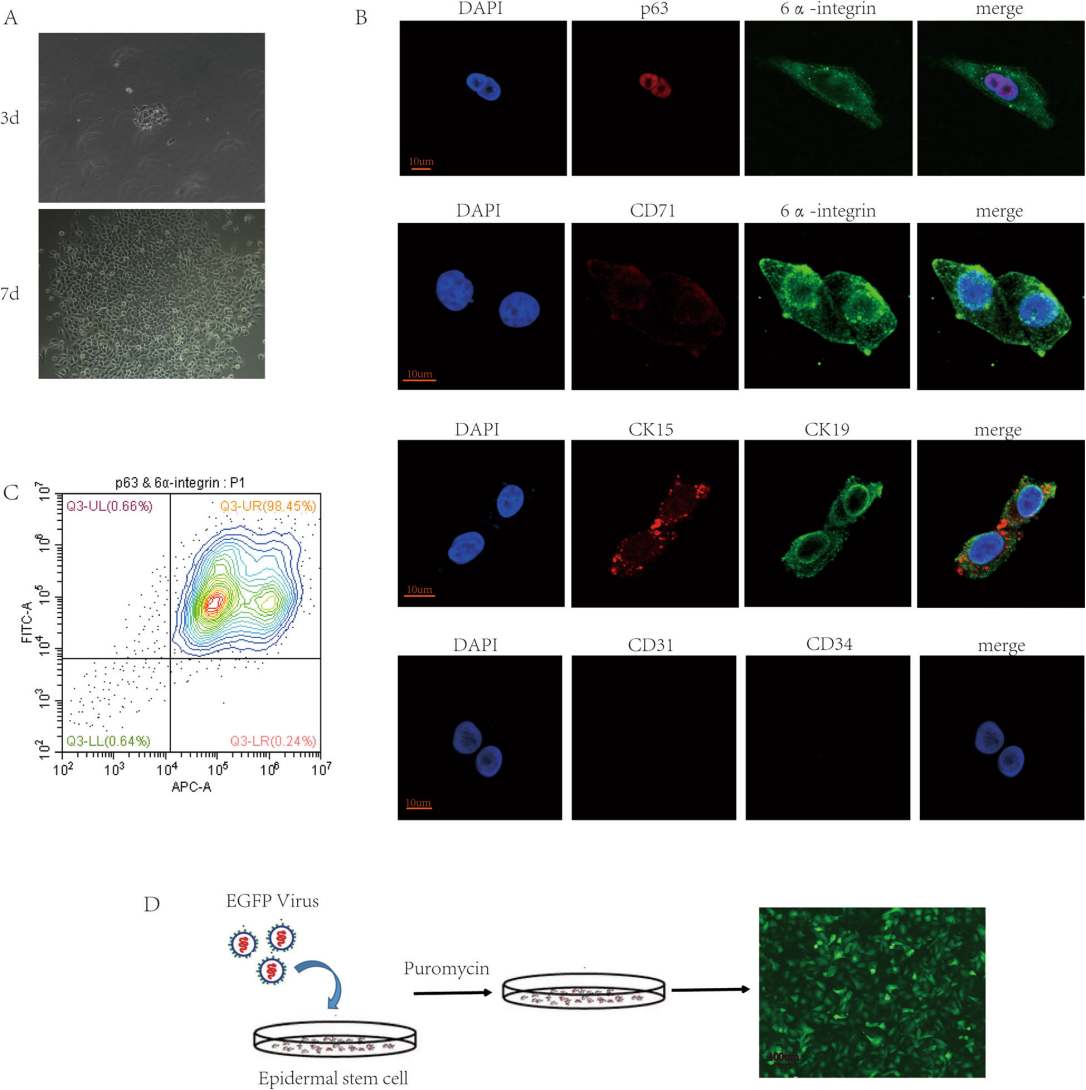
Isolation, identification, and lentivirus transfection of rat ESCs. **a** Rat ESC morphology on the 3rd and 7th days (× 10 microscope). **b** Identification of third-generation cells using p63, 6α integrin, CD71, Ck15, CK19, CD31, and CD34. **c** Flow cytometry to detect the proportion of p63- and 6α integrin-positive cells. **d** Rat ESCs were transfected with lentivirus to stably express EGFP

Our previous study found that FN-precoated culture dishes promote the adhesion and proliferation of ESCs [14]. In our study, we used FN to harvest and expand rat ESCs. The isolated original representative skin stem cells were round in shape, small in size, and strong in refraction. After overnight culture, some cells adhered to the wall; these cells were polygonal, and the nuclei were larger. After culturing rat ESCs for 3 days and changing the medium, the cells formed a clonal colony and adhered firmly. After 7 days of culture, the cells proliferated significantly, and they were connected in a paving-stone-like sheet shape (Fig. 1a). Rat ESCs were passaged and expanded, and third-generation cells were analyzed for immunofluorescence identification. The cells expressed p63 and 6α integrin, as well as CD71dim. Moreover, CK15 and CK19 were positive in these cells. Although CD31 and CD34 were negative (Fig. 1b), the isolated cells were ESCs but not vascular endothelial cells. The results of cell flow cytometry analysis indicated that the p63- and 6α integrin-positive cells accounted for approximately 98.53% of the third-generation cells (Fig. 1c).

### Rat ESCs improve wound closure and the healing quality of SD rats

**Fig. 2:**
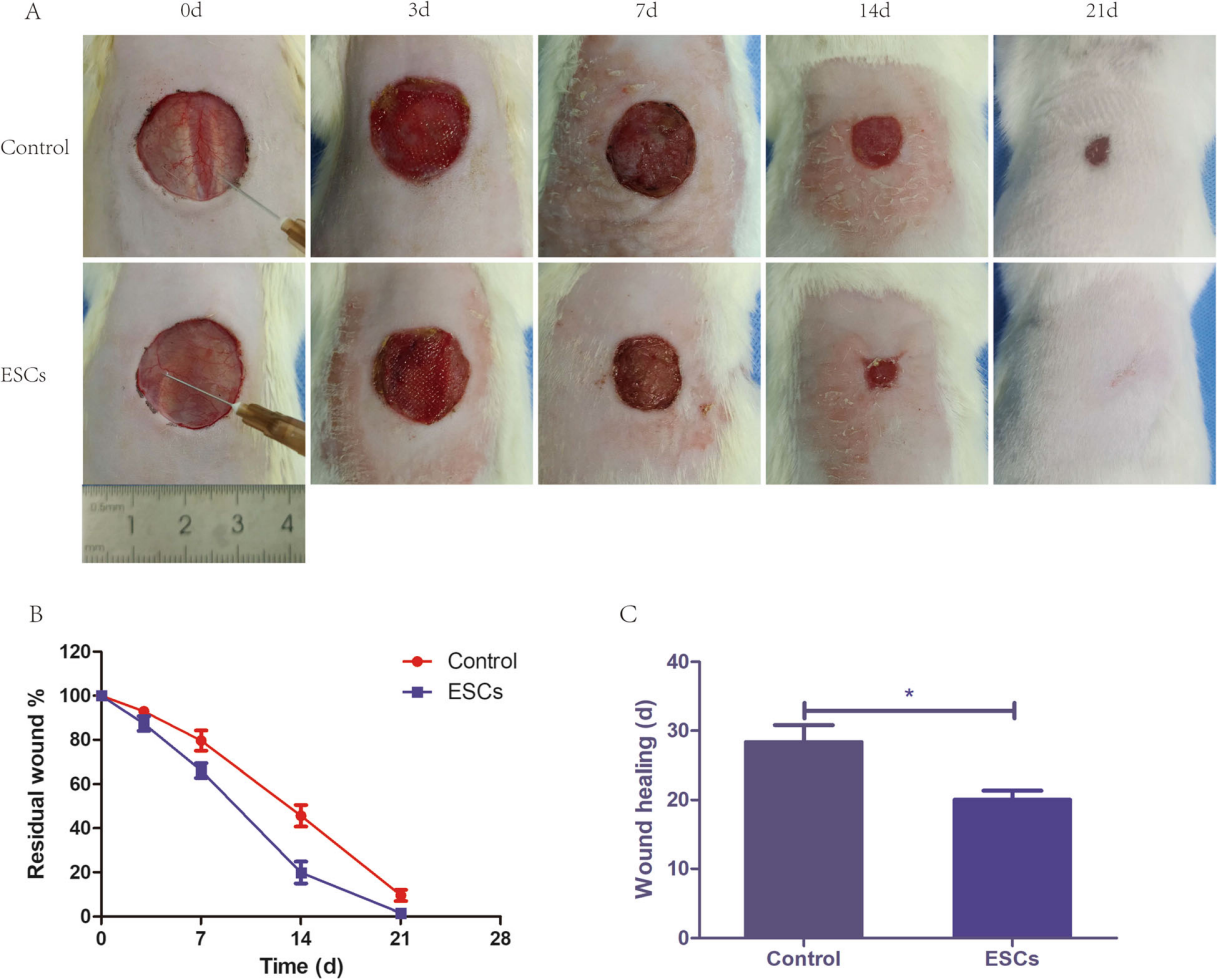
Rat ESCs accelerate wound closure and improve the healing quality of rats. **a** The wound pictures of the rats’ dorsal skin of the negative control group and ESC group were taken on postinjury days 0, 3, 7, 14, and 21. **b** Residual wound rates of the negative control group and ESC group on postinjury days 0, 3, 7, 14, and 21. **c** Completed wound healing time of the negative control group and ESC group. *P < 0.05

Third-generation rat ESCs were transfected with lentivirus to express EGFP (Fig. 1d). We used the ESCs that stably expressed EGFP for subsequent animal experiments. To investigate whether ESCs can influence the healing of the wound bed in vivo, we used the rat dorsal wound model. Compared with the control group, the ESC group displayed a dramatically higher healing quality (Fig. 2a), a lower residual wound area (Fig. 2b), and a shorter healing time (Fig. 2c). These results suggested that ESCs spray could promote wound healing and improve the healing quality significantly.

### Rat ESCs promote the angiogenesis of wounds

**Fig. 3:**
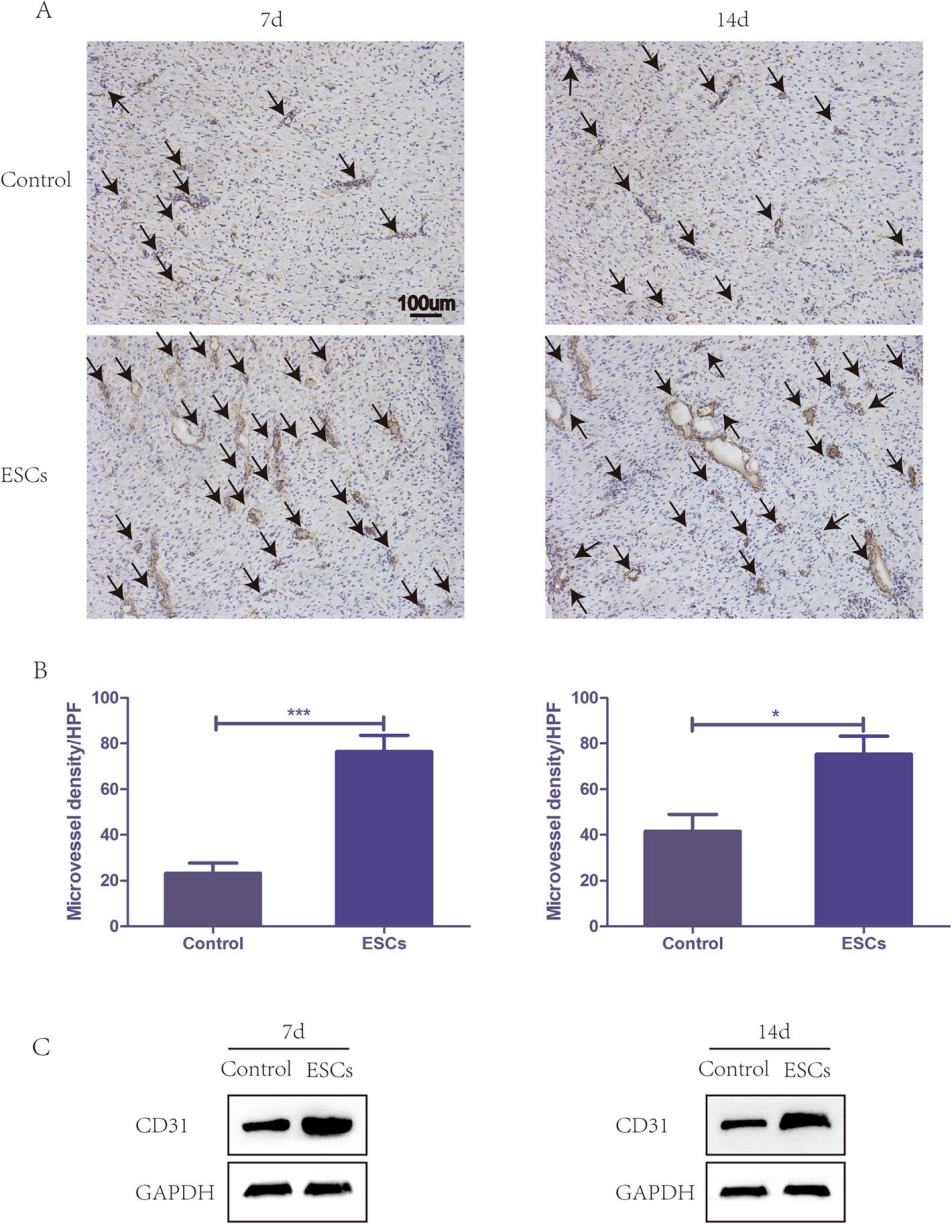
Expression of angiogenesis factors in rat wounds on days 7 and 14. **a**, **b** Representative area and analysis of wound tissue sections stained with CD31 on postinjury days 7 and 14 showing microvascular regeneration in rat wounds in the negative control group and ESC group. Arrows indicate the microvascular. **c** Representative western blot analysis showing the relative protein levels of CD31 for each group on days 7 and 14. ***P < 0.0001, *P < 0.05

To assess angiogenesis, the wound area sections on days 7 and 14 were stained with CD31 for immunohistochemistry. Microvessel density (MVD) was assessed using CD31-positive cells in five areas randomly. The ESC group displayed significantly higher MVD than the control group at both time points, and CD31 expression was strongly positive (Fig. 3a, b). Similarly, western blots of wound snap-frozen samples on days 7 and 14 also showed a markedly higher CD31 expression in the ESC group than that in the control group (Fig. 3c). All the results above demonstrated that ESCs could improve wound healing by accelerating angiogenesis.

### Rat ESCs could differentiate into vascular endothelial cells

**Fig. 4:**
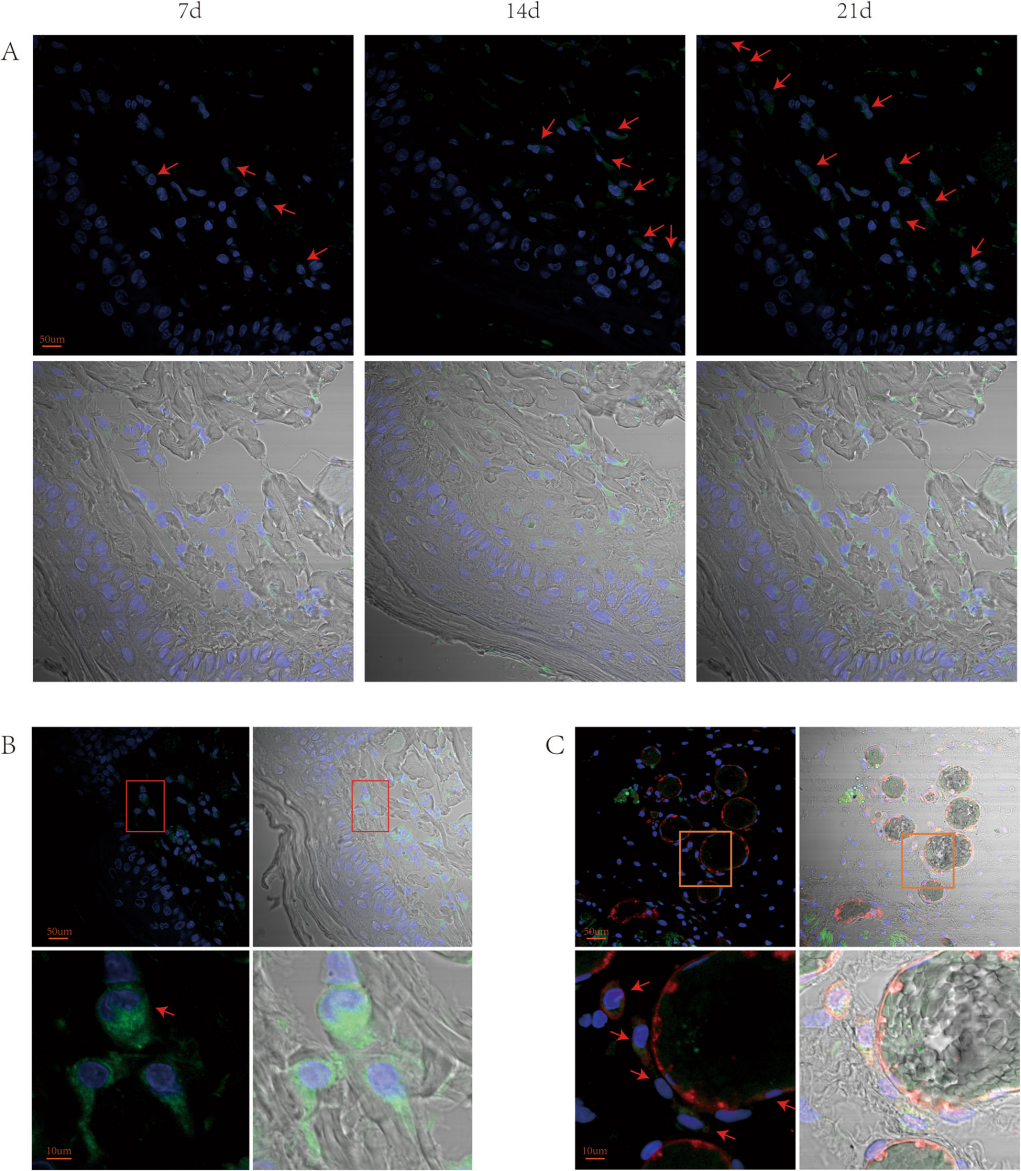
Tracking rat ESCs in the wound on postinjury day 14. **a** Rat ESCs expressing EGFP were observed in the wound on days 7, 14, and 21 after the operation. Arrows indicate the EGFP-positive cells. **b** The rat ESCs have dividing and proliferating phases. **c** The rat ESCs near blood vessels can express CD31 and participate in the formation of lumen-like structures. Arrows indicate the CD31- and EGFP-positive cells

To explore the role of rat ESCs sprayed on the wound surface in the process of wound healing, we have transfected rat ESCs with lentivirus in our previous experiments to stably express ESCs. We took tissue samples from the wounds of rats for immunofluorescence staining. On the 7th, 14th, and 21st days, ESCs that stably expressed EGFP could be observed on the wounds of rats (Fig. 4a). The ESCs were mainly colonized in the subcutaneous tissue layer, and the ESCs divided and proliferated (Fig. 4b). Immunofluorescence staining of blood vessels with CD31 revealed red staining in vascular endothelial cells—that is, CD31 expression was positive, and ESCs can be seen in CD31-positive cells (Fig. 4c). The above results showed that the ESCs sprayed on the wound surface can be colonized on wounds to divide and proliferate and can differentiate toward vascular endothelial cells to form a lumen structure.

## Discussion

Various factors can cause skin wounds, and the types and locations of wounds caused by different events may also be different. Thus, the treatment of wounds is various and difficult. Moreover, wounds may affect the life quality of patients to varying degrees and increase the social medical burden. Among the factors that cause wound nonhealing, vascular regeneration disorder is a key factor. Therefore, a large amount of research is currently focused on vascular regeneration to promote wound healing by enhancing angiogenesis. Many studies have reported that increasing wound angiogenesis can significantly promote wound repair [17–21]. However, because wound repair is a pathophysiological process involving multiple factors, any unbalanced factors will cause abnormal wound healing. Therefore, wound repair remains a common problem worldwide.

In recent years, stem cell therapy has brought a new dawn for wound repair because stem cells have promising characteristics, such as the ability to differentiate multidirectionally and secrete paracrine growth factors. Adult stem cells are favored by scholars because of their immunocompatibility and ethical constraints. ESCs play an important role in skin repair because ESCs can self-proliferate and differentiate, promote wound healing, and restore normal epidermal structure and skin function [22–24]. A study found that Rhesus putative ESCs can trans-differentiate into corneal epithelium-like cells when cocultured with human corneal limbal stroma and corneal epithelial cells [25]. Thus, ESCs may have the potential for multidirectional differentiation under certain conditions.

In our study, we successfully isolated and expanded rat ESCs in vitro to treat wounds. The results showed that rat ESCs could promote the healing of full-thickness skin defects in rats. Moreover, we found that rat ESCs can promote angiogenesis in the wound bed of the rats. In that experiment, we transfected the rat ESCs with lentivirus to stably express EGFP, which we expanded in vitro. Thus, we could track the ESCs sprayed on the wound bed. On the 21st day after the operation, ESCs that stably expressed EGFP could still be observed on the wounds of the rats. The ESCs were mainly colonized in the subcutaneous tissue layer and proliferated in the wound bed. Furthermore, the ESCs near the blood vessels expressed CD31, which is a marker of vascular endothelial cells. Thus, ESCs can differentiate into vascular endothelial cells. Some ESCs were observed around the vessels to form vascular tubes. Therefore, we speculated that rat ESCs in the vicinity of blood vessels could differentiate into vascular endothelial cells and participate in the formation of lumen-like structures, indicating that rat ESCs might have partial functions in vascular endothelial cells.

## Conclusion

In summary, our study showed that rat ESCs could promote the formation of new blood vessels and accelerate wound healing in full-thickness skin defects in rats. Regarding the mechanism, rat ESCs can colonize and proliferate in the wound bed, and the rat ESCs near the blood vessels can differentiate into vascular endothelial cells to promote angiogenesis. Our study provided a theoretical basis for ESCs to treat full-thickness skin defect wounds and proved that ESCs are safe and effective in treating wounds even after in vitro expansion. We found that ESCs can differentiate into vascular endothelium cells and can form vessel tubes under certain conditions. The mechanism of the effect of ESCs on wound healing and the multidirectional differentiation potential of ESCs warrant further study to understand the therapeutic effects of ESCs and expand their therapeutic scope for diseases.

## Data Availability

The data that support the findings of this study are available from the corresponding author upon reasonable request.

## Abbreviations

ESCs: Epidermal stem cells
FN: Fibronectin
PBS: Phosphate buffered saline
K-SFM: Keratinocyte serum-free medium
EGFP: Enhanced green fluorescent protein
BCA: Bicinchoninic acid assay kit
CD31: Cluster of differentiation 31
CD34: Cluster of differentiation 34
GAPDH: Glyceraldehyde-3-phosphate dehydrogenase
IF: Immunofluorescence
IHC: Immunohistochemistry
WB: Western blot
MVD: Microvessel density
